# Publisher correction: Functional characterization of a multi-cancer risk locus on chr5p15.33 reveals regulation of *TERT* by ZNF148

**DOI:** 10.1038/ncomms16159

**Published:** 2018-03-05

**Authors:** Jun Fang, Jinping Jia, Matthew Makowski, Mai Xu, Zhaoming Wang, Tongwu Zhang, Jason W. Hoskins, Jiyeon Choi, Younghun Han, Mingfeng Zhang, Janelle Thomas, Michael Kovacs, Irene Collins, Marta Dzyadyk, Abbey Thompson, Maura O'Neill, Sudipto Das, Qi Lan, Roelof Koster, Rachael S. Stolzenberg-Solomon, Peter Kraft, Brian M. Wolpin, Pascal W. T. C. Jansen, Sara Olson, Katherine A. McGlynn, Peter A. Kanetsky, Nilanjan Chatterjee, Jennifer H. Barrett, Alison M. Dunning, John C. Taylor, Julia A. Newton-Bishop, D Timothy Bishop, Thorkell Andresson, Gloria M. Petersen, Christopher I. Amos, Mark M. Iles, Katherine L. Nathanson, Maria Teresa Landi, Michiel Vermeulen, Kevin M. Brown, Laufey T. Amundadottir

Nature Communications 8: Article number: 15034; DOI: 10.1038/ncomms15034 (2017); Published: 05 27 2017; Updated: 03 05 2018

The original version of this Article contained an error in the spelling of two members of the GenoMEL Consortium, Joan Anton Puig-Butille and Pol Gimenez-Xavier, which were incorrectly given as Joan-Anton Puig Butille and Pol Gimenez Xavier respectively. Furthermore, the PDF version of the Article also contained an error in the publication date, which was incorrectly given as 2nd May 2017. These errors have now been corrected in both the PDF and HTML versions of the Article.

**PanScan Consortium**

Federico Canzian, Charles Kooperberg, Zhaoming Wang, Alan A. Arslan, Paige M. Bracci, Julie Buring, Eric J. Duell, Steven Gallinger, Eric J. Jacobs, Aruna Kamineni, Stephen Van Den Eeden, Alison P. Klein, Laurence N. Kolonel, Donghui Li, Sara H. Olson, Harvey A. Risch, Howard D. Sesso, Kala Visvanathan, Wei Zheng, Demetrius Albanes, Melissa A. Austin, Marie-Christine Boutron-Ruault, H. Bas Bueno-de-Mesquita, Michelle Cotterchio, J. Michael Gaziano, Edward L. Giovannucci, Michael Goggins, Myron Gross, Manal Hassan, Kathy J. Helzlsouer, Elizabeth A. Holly, David J. Hunter, Mazda Jenab, Rudolf Kaaks, Timothy J. Key, Kay-Tee Khaw, Vittorio Krogh, Robert C. Kurtz, Andrea LaCroix, Loic Le Marchand, Satu Mannisto, Alpa V. Patel, Petra H.M. Peeters, Elio Riboli, Xiao-Ou Shu, Malin Sund, Mark Thornquist, Anne Tjønneland, Geoffrey S. Tobias, Dimitrios Trichopoulos, Jean Wactawski-Wende, Herbert Yu, Kai Yu, Anne Zeleniuch-Jacquotte, Robert Hoover, Patricia Hartge, Charles Fuchs & Stephen J. Chanock

**TRICL Consortium**

Victoria Stevens, Demetrios Albanes, Neil E. Caporaso, Paul Brennan, James McKay, Xifeng Wu, Rayjean J. Hung, John R. McLaughlin, Heike Bickeboller, Angela Risch, Erich Wichmann & Richard S. Houlston

**GenoMEL Consortium**

Graham Mann, John Hopper, Joanne Aitken, Bruce Armstrong, Graham Giles, Elizabeth Holland, Richard Kefford, Anne Cust, Mark Jenkins, Helen Schmid, Susana Puig, Paula Aguilera, Celia Badenas, Alicia Barreiro, Cristina Carrera, Daniel Gabriel, Pol Gimenez-Xavier, Pablo Iglesias-Garcia, Josep Malvehy, Montse Mila, Ramon Pigem, Miriam Potrony, Joan Anton Puig-Butille, Gemma Tell-Marti, Nicholas K. Hayward, Nicholas G. Martin, Grant Montgomery, David L. Duffy, David C. Whiteman, Stuart MacGregor, Donato Calista, Giorgio Landi, Paola Minghetti, Fabio Arcangeli, Pier Alberto Bertazzi, Paola Ghiorzo, Giovanna Bianchi-Scarra, Lorenze Pastorino, William Bruno, Virginia Andreotti, Paola Queirolo, Francesco Spagnolo, Rona MacKie, Julie Lang, Nelleke Gruis, Frans A. van Nieuwpoort, Coby Out, Wilma Bergman, Nicole Kukutsch, Jan Nico Bouwes Bavinck, Bert Bakker, Nienke van der Stoep, Jeanet ter Huurne, Han van der Rhee, Marcel Bekkenk, Dyon Snels, Marinus van Praag, Lieve Brochez, Rianne Gerritsen, Marianne Crijns, Hans Vasen, Bart Janssen, Christian Ingvar, Håkan Olsson, Göran Jönsson, Åke Borg, Katja Harbst, Kari Nielsen, Anita Schmidt Zander, Anders Molven, Per Helsing, Per Arne Andresen, Helge Rootwelt, Lars A. Akslen, Brigitte Bressac-de Paillerets, Florence Demenais, Marie-Francoise Avril, Valerie Chaudru, Patricia Jeannin, Fabienne Lesueur, Eve Maubec, Hamida Mohamdi, Myriam Bossard, Amaury Vaysse, Francoise Boitier, Olivier Caron, Frederic Caux, Stephane Dalle, Olivier Dereure, Dominique Leroux, Ludovic Martin, Christine Mateus, Caroline Robert, Dominique Stoppa-Lyonnet, Luc Thomas, Eva Wierzbicka, David E. Elder, Michael Ming, Nandita Mitra, Tadeusz Debniak, Jan Lubinski, Marko Hocevar, Srdjan Novakovic, Barbara Peric, Petra Skerl, Johan Hansson, Veronica Höiom, Eitan Friedman, Esther Azizi, Orna Baron-Epel, Alon Scope, Felix Pavlotsky, Irit Cohen-Manheim, Yael Laitman, Mark Harland, Juliette Randerson-Moor, Jon Laye, John Davies, Jeremie Nsengimana, Sally O’Shea, May Chan, Jo Gascoyne, Margaret A. Tucker, Alisa M. Goldstein & Xiaohong R. Yang

