# Supplementary material for: Functional characterization of a multi-cancer risk locus on chr5p15.33 reveals regulation of TERT by ZNF148
Source: Nat Commun. 2017 Apr 27;8:15034. doi: 10.1038/ncomms15034 (PMC5414179; doi:10.1038/ncomms15034)
Supplement: Supplementary Information — Supplementary Figures and Supplementary Tables [file ncomms15034-s1.pdf]

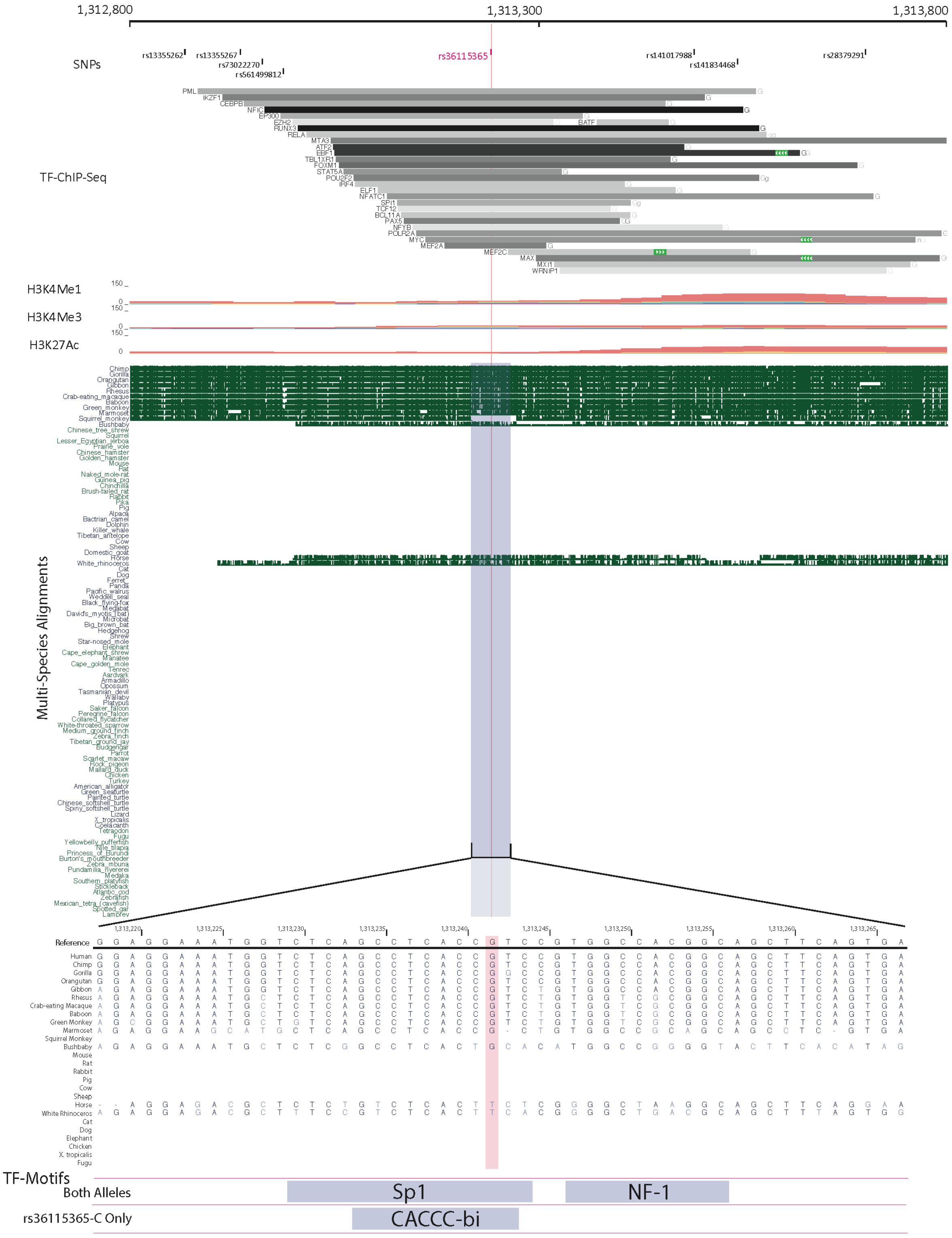

Supplementary Figure 1 | Multi-species conservation and predicted transcription factor motifs for the genomic region surrounding rs36115365. Transcription factor chromatin immunoprecipitation sequencing (TF-ChIP-Seq), layered H3K4Me1, H3K4Me3, and H3K27Ac ChIP-seq, and multi-species conservation is shown (top panel). Multi-species conservation, as well as predicted transcription factor binding motifs (Alibaba 2.1) to the reference and rs36115365-C variant alleles are shown (bottom panel). CACCC-bi: CACCC-binding protein DNA recognition motif known to bind transcription factors of the kruppel-like family.

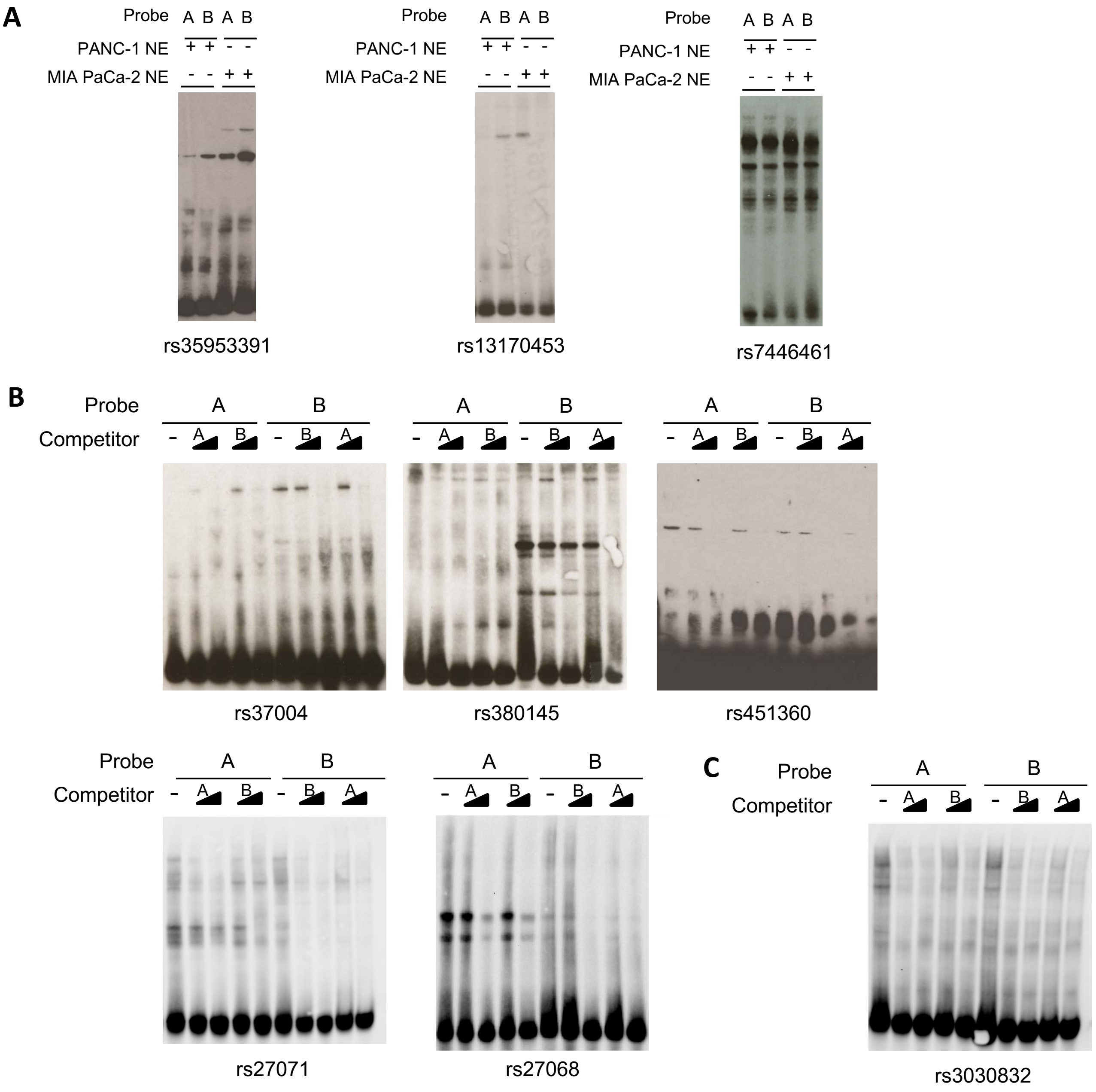

Supplementary Figure 2 | EMSA for 9 variants highly correlated to rs36115365. **a,** Allelic differences in protein binding were not seen for rs35953391, rs13170453 and rs7446461. **b,** Suggestive evidence for differences in binding to the two alleles were noted but competition with un-labelled oligonucleotides showed that either both (rs37004, rs451360, rs27071 and rs27068) or neither (rs380145) of the competitors competed for binding (PANC-1 nuclear extract used for all SNPs) indicating unspecific binding. Allele designation: rs35953391 A/B is T/C; rs13170453 A/B is A/G; rs7446461 A/B is G/C; rs37004 A/B is T/C; rs380145 A/B is T/C; rs451360 A/B is T/G; rs27071 A/B is T/C, rs27068 A/B is C/T (red letters indicate minor alleles which are also risk alleles for pancreatic cancer and testicular germ cell tumors). **c,** EMSA (using PANC-1 nuclear extract) for an indel variant that was not included in the association analysis, and, is called in a newer 1000G version (phase 3, version 1) and is highly correlated to rs36115365 (r^2=0.87 in EUR). A/B for rs3030832 is C/CTG. Allele specific differences in protein binding were not seen for this variant.

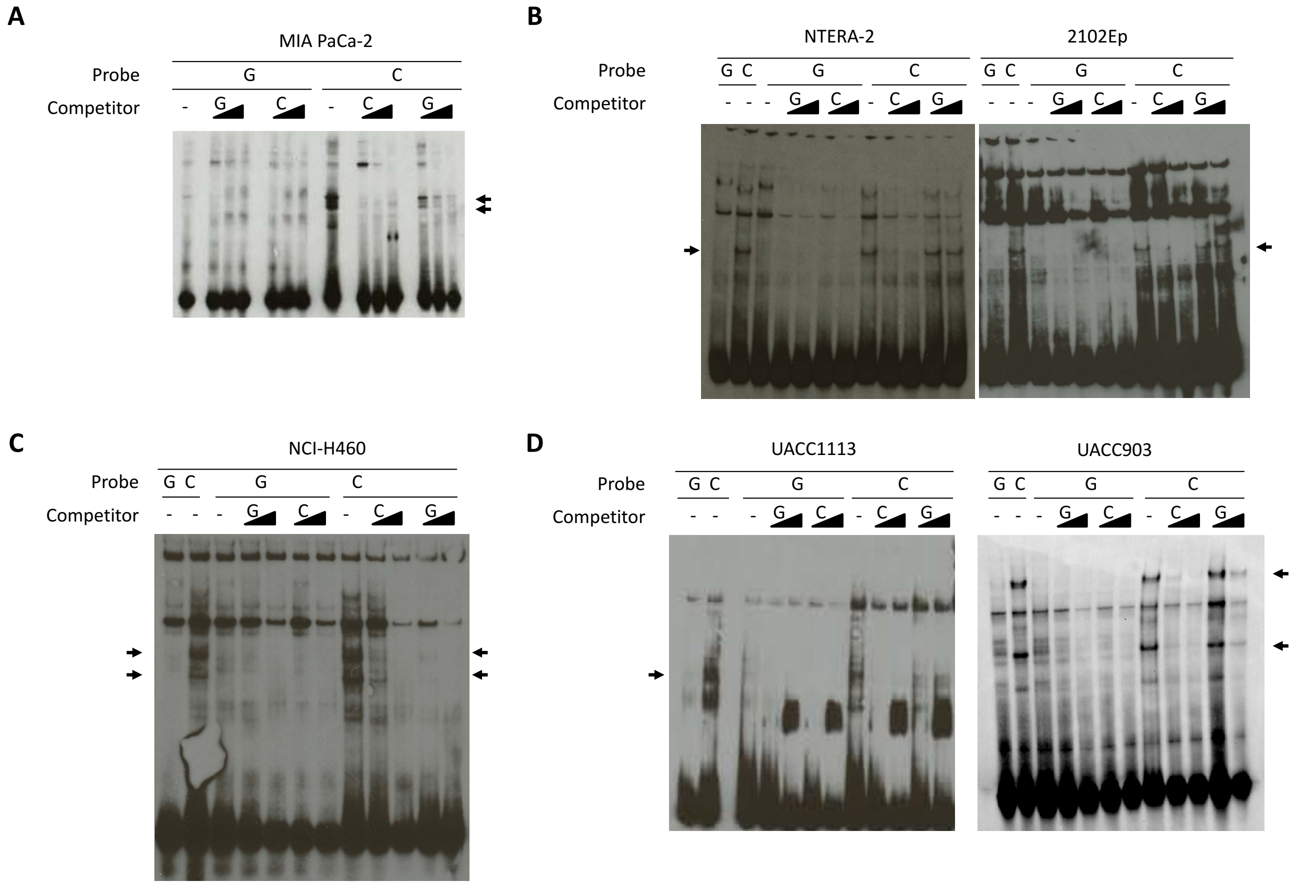

Supplementary Figure 3 | EMSA for rs36115365 in six cell lines. | a, Pancreatic cancer cell line (MIA PaCa-2). **b,** Testicular germ cell tumor cell lines (NTERA-2 and 2102Ep). **c**, Lung cancer cell line (NCI-H460). **d**, Melanoma cell lines (UACC1113, UACC903). C/G indicates genotype at rs36115365 for the probe used. Unlabeled competitor was used at 10x, 50x and 100x (as indicated by gradient symbol) to assess specificity of protein binding to the labeled probe.

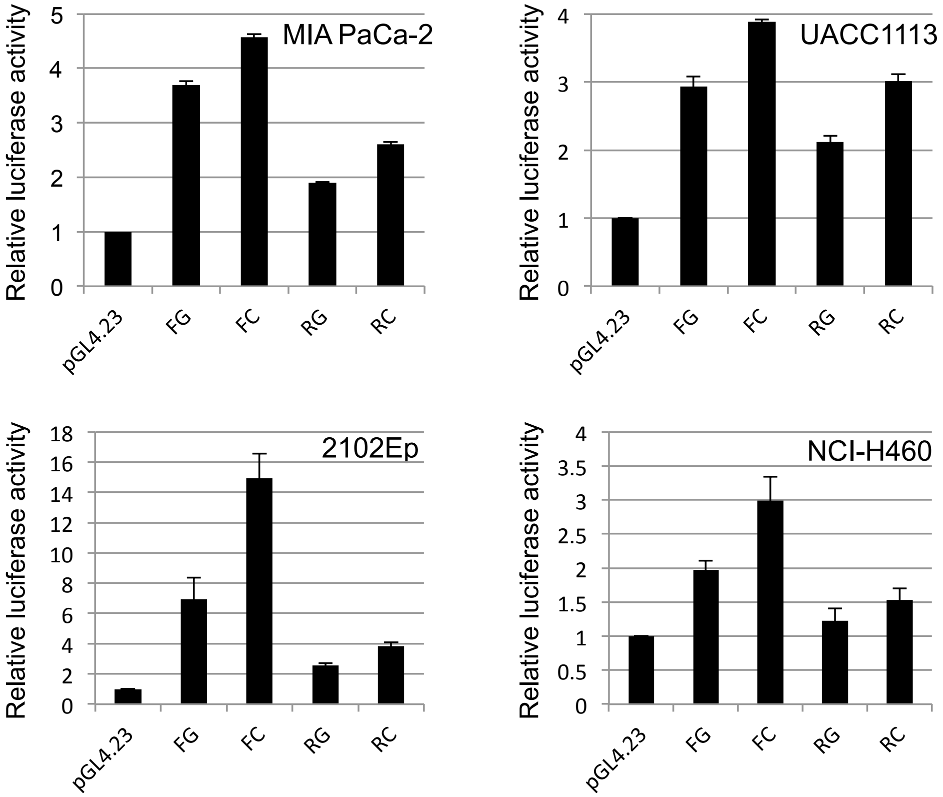

Supplementary Figure 4 | Allele specific luciferase activity for rs36115365 in four cancer derived cell lines from pancreas (MIA PaCa-2), melanoma (UACC1113), testis (2102Ep) and lung (NCI-H460). DNA fragments containing the different alleles (C/G) of rs36115365 were cloned into pGL4.23 in the forward (F) and reverse (R) orientation before transient transfection and luciferase assays were performed. Luciferase activity is shown relative to the empty vector (pGL4.23) with standard error of the mean (SEM) for at least three experiments per cell line.

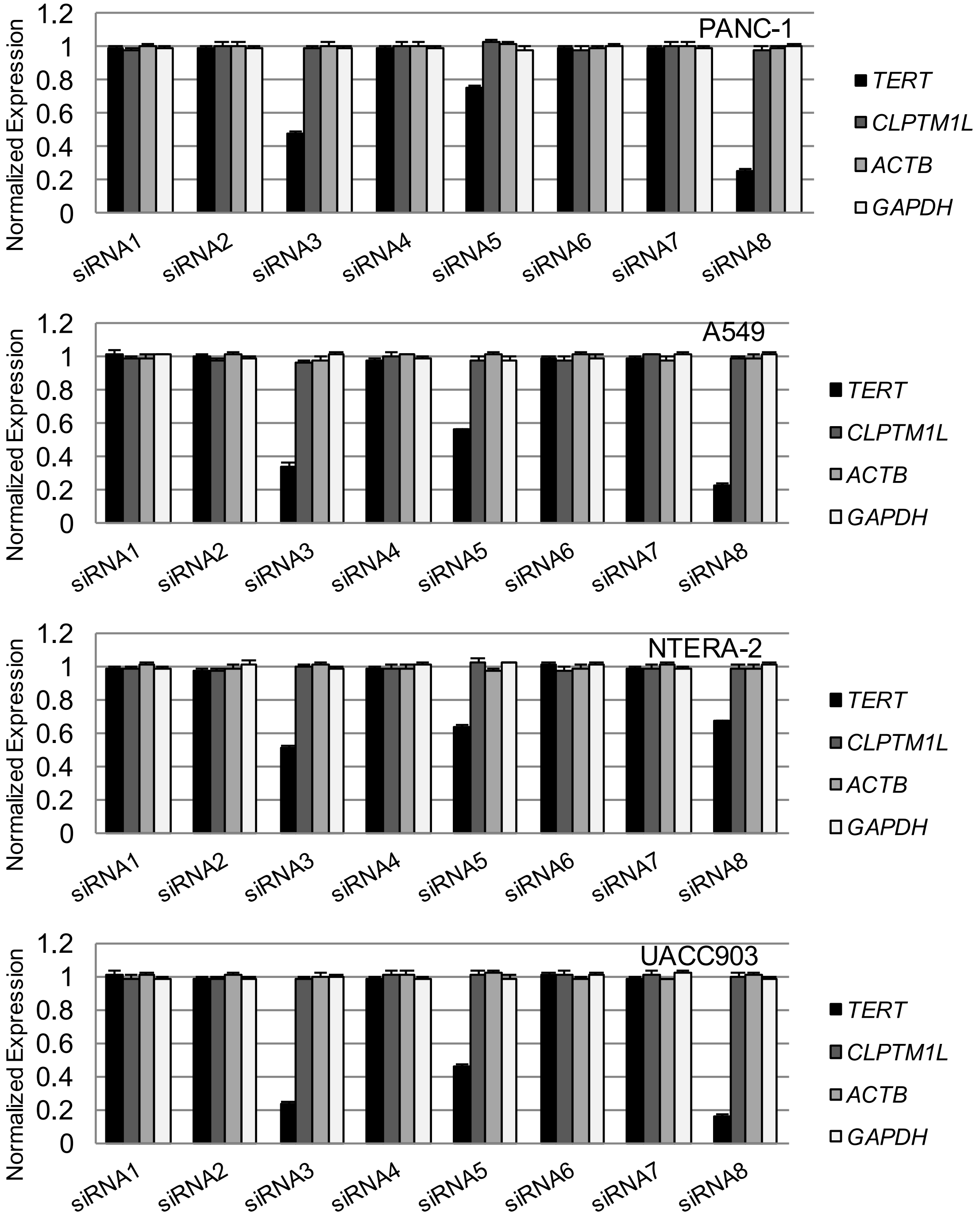

Supplementary Figure 5 | Targeted siRNA inhibition of potential gene regulatory region encompassing rs36115365 reduces gene expression of *TERT* but not *CLPTM1L*. Eight double-strand siRNAs were designed to target the potential gene-regulatory region encompassing rs36115365. siRNAs were transfected into PANC-1 (pancreas), A549 (lung), NTERA-2 (testis), or UACC903 (melanoma) cell lines and expression levels of *TERT*, *CLPTM1L*, and two control genes (*ACTB* and *GAPDH*) were quantitated relative to those from cells transfected with a scrambled siRNA control. Three of eight double-strand siRNAs targeting rs36115365 locus inhibited the gene expression of *TERT*, but none altered levels of *CLPTM1L*, *ACTB*, and *GAPDH*. Experiments were performed in triplicate and repeated three times; errors bars represent standard error of the mean (SEM) across three separate experiments.

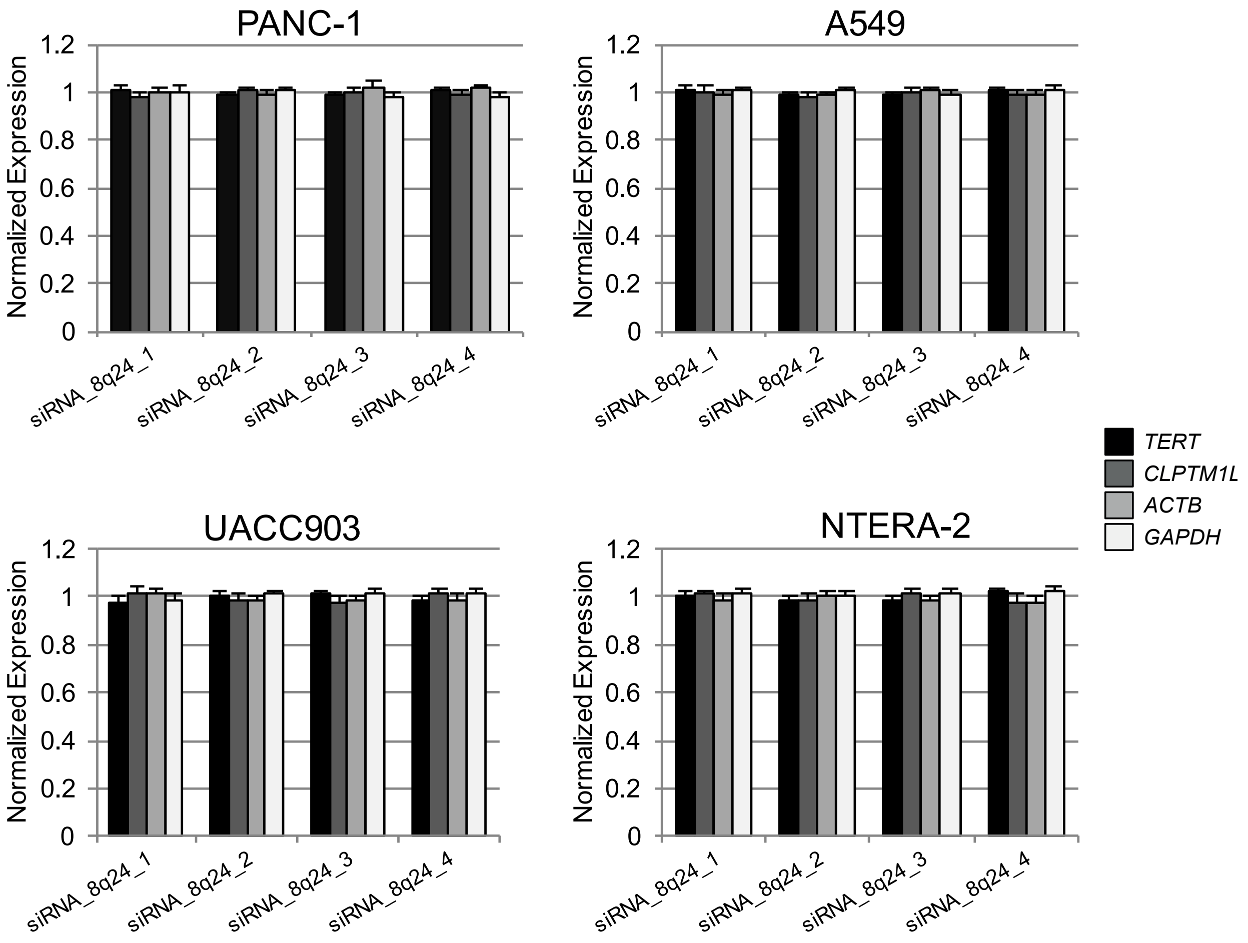

Supplementary Figure 6 | Region-targeted siRNA inhibition using control siRNAs designed against chromosome 8q24.21 do not inhibit expression of *TERT* or *CLPTM1L*. Double-strand siRNAs were designed to target potential gene-regulatory regions on chromosome band 8q24.21. siRNAs were transfected into PANC-1 (pancreas), A549 (lung), NTERA-2 (testis), or UACC903 (melanoma) cell lines and expression levels of *TERT*, *CLPTM1L*, and two control genes (*ACTB* and *GAPDH*) were quantitated relative to those from cells transfected with a scrambled siRNA control. None of the siRNAs significantly altered expression of any of the genes assayed. Experiments were performed in triplicate and repeated three times; errors bars represent standard error of the mean (SEM) across three separate experiments.

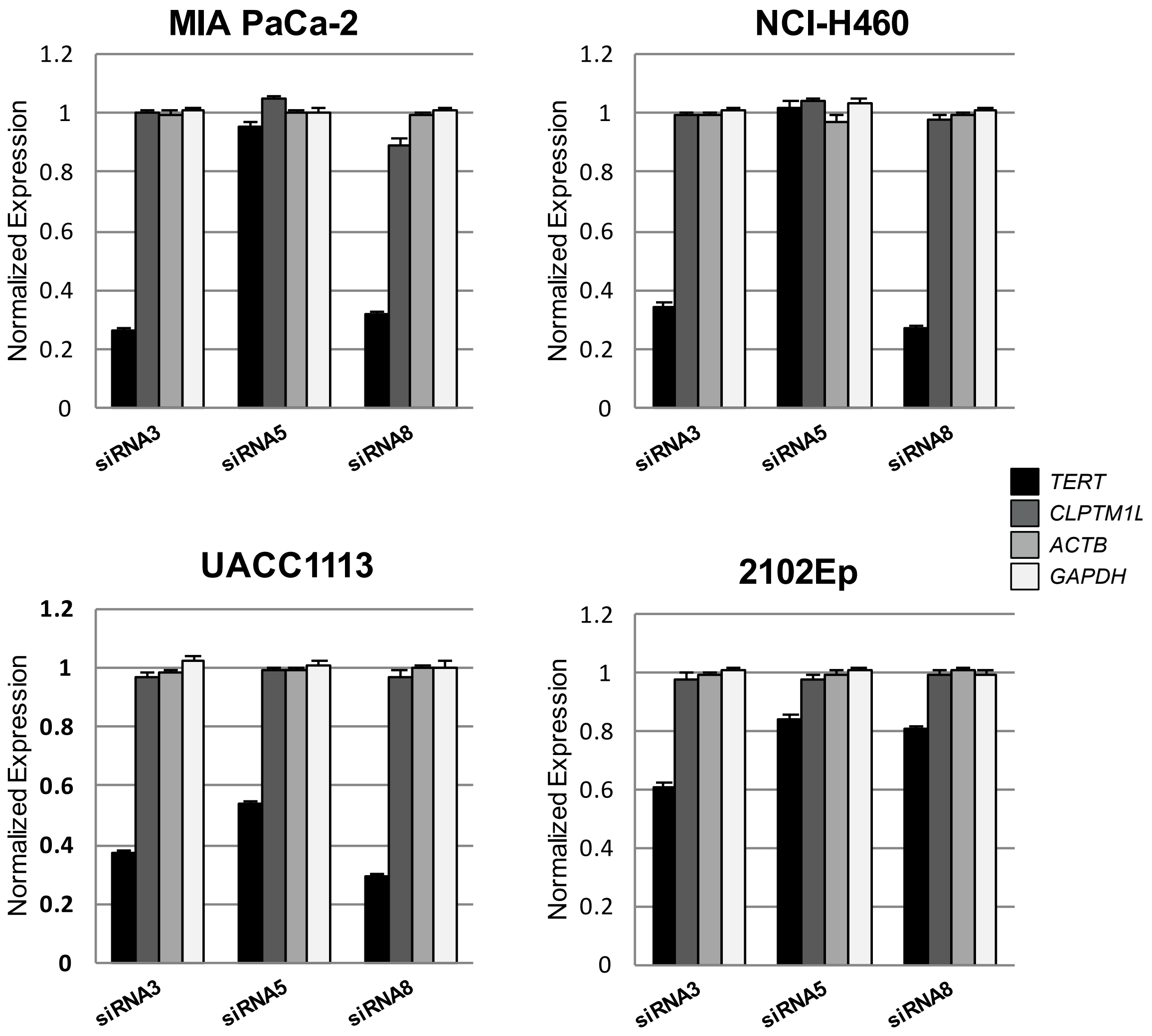

Supplementary Figure 7 | Targeted siRNA inhibition of potential gene regulatory region encompassing rs36115365 reduces gene expression of *TERT* but not *CLPTM1L*. Three of eight double-strand siRNAs targeting the rs36115365 locus inhibit gene expression of *TERT* but not *CLPTM1L*, *ACTB* and *GAPDH* in MIA PaCa-2 (pancreatic cancer), NCI-H460 (lung cancer), UACC1113 (melanoma) and 2102Ep (testicular cancer). The gene expression was normalized to *GAPDH* except that gene expression of *GAPDH* was normalized to *ACTB.* Error bars represent standard error of the mean (SEM) across three separate experiments.

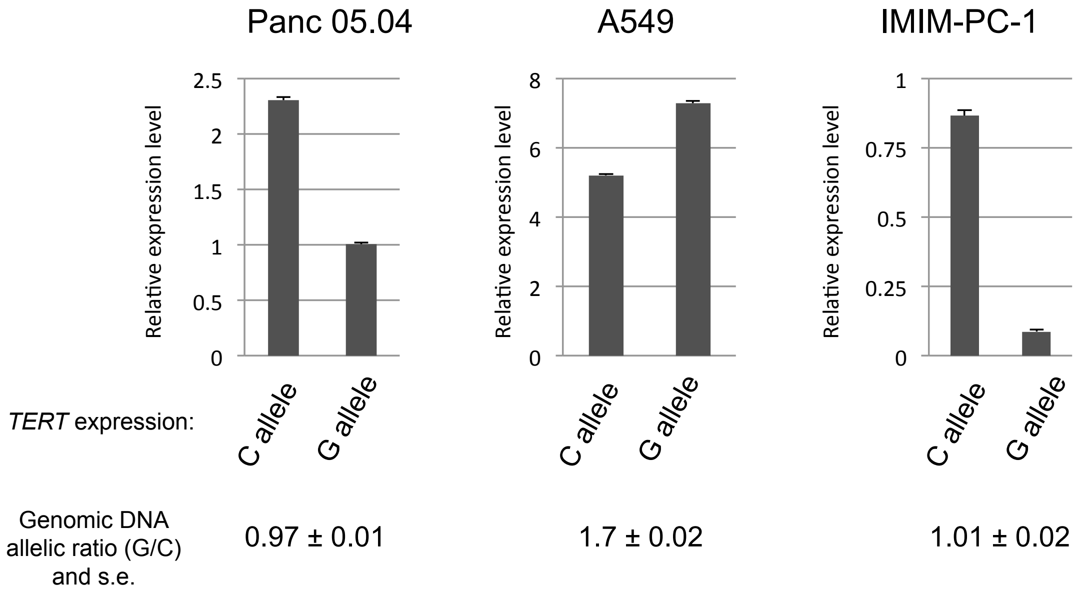

Supplementary Figure 8 | TERT expression from the C and G allele at rs36115365. Allele specific *TERT* expression from rs36115365 was quantified using a quantitative allele-discrimination TaqMan assay for a synonymous coding SNP in the *TERT* gene (rs2736098, r^2=0.14, D'=1.0). Two pancreatic cancer cell lines (Panc 05.04 and IMIM-PC-1) and one lung cancer cell line (A549) were used as they are heterozygous for both SNPs. Relative expression levels from cDNA were normalized to 1 ng genomic DNA from a HapMap individual heterozygous for rs2736098. Error bars represent standard error of the mean (SEM) across three separate experiments. The G/C allelic ratio for rs2736098 at the genomic DNA level was measured using an allelic discrimination assay, standard error of the mean (SEM) across three separate experiments is shown.

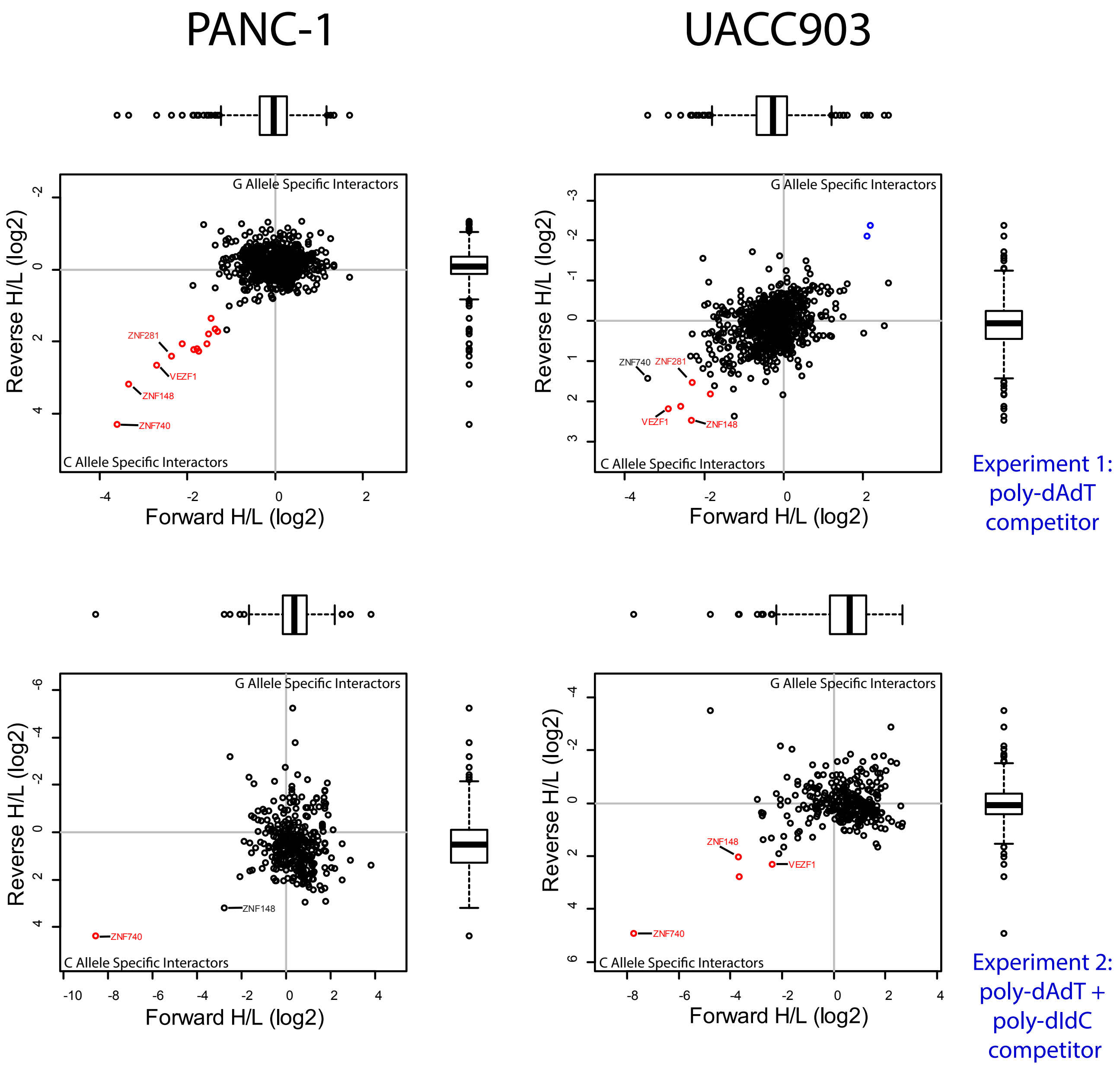

Supplementary Figure 9 | Quantitative mass-spectrometric identification of allele-specific binding proteins to rs36115365. DNA pulldowns were performed using PANC-1 (pancreatic cancer) and UACC903 (melanoma) cell line nuclear extracts with 41bp biotin-tagged rs36115365 bait oligos. Replicate label-swapping experiments were performed both using poly-dAdT competitor only (top), or both poly-dAdT and poly-dIdC competitors. Ratios indicate enrichment for protein binding to either the C- or G-allele. Significantly enriched ratios in both a forward and reverse label-swapping experiment were called as outliers (Red: C-allele, Blue: G-allele. Outlier cutoff is 1.5 IQRs). The identities of C-specific binding proteins consistently identified as outliers across cell lines in either poly-dAdT or mixed poly-dAdT/poly-dIdC experiments are noted. Boxes in boxplots represent first to third quartiles and whiskers extend to furthest data point still within 1.5 IQRs of either quartile.

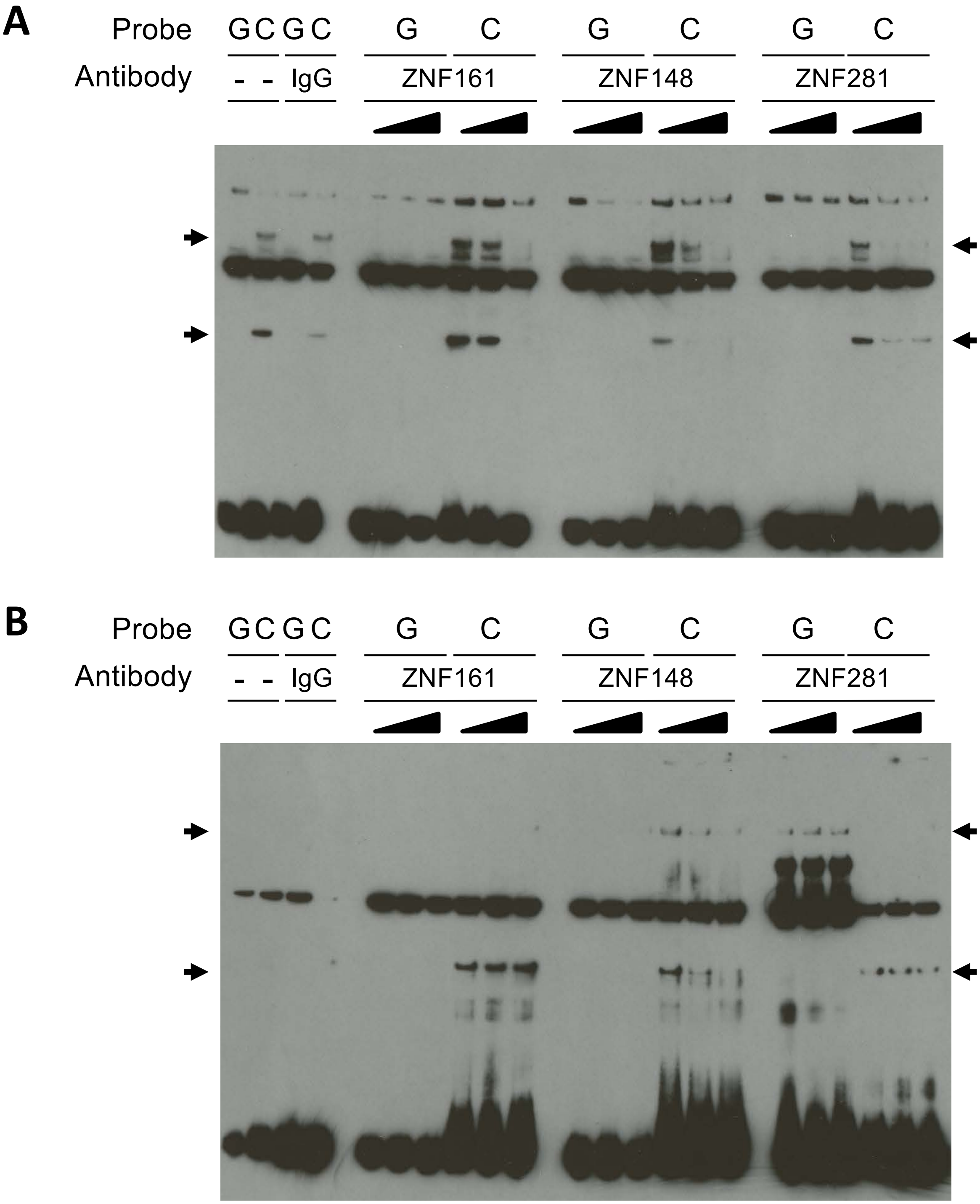

Supplementary Figure 10 | EMSA and supershift analysis for three Zn finger proteins noted by proteomics analysis to bind rs36115365 C-allele. a, Lung cancer cell line (A549). **b**, Testicular germ cell tumor cell line (NTERA-2). C/G indicates genotype at rs36115365 for the probe used. Antibodies to VEZF1/ZNF161, ZNF148/ZBP-89 and ZNF281 were used at 1 mg and 2 mg (as indicated by gradient symbol) to compete for binding of the respective proteins to the probes. IgG was used as a negative control antibody at 2 mg per reaction.

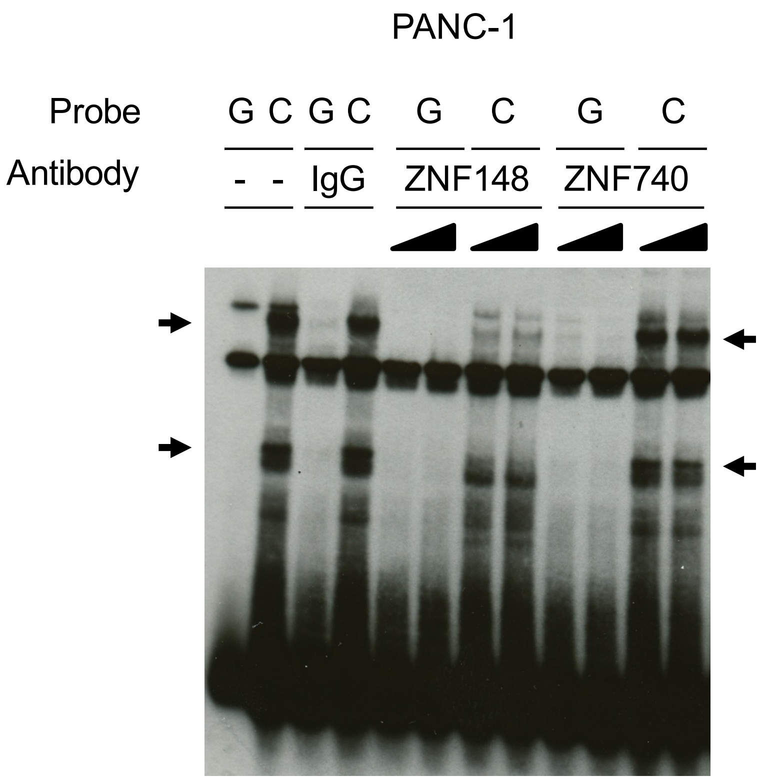

Supplementary Figure 11 | EMSA and supershift for rs36115365 with ZNF148 and ZNF740 in PANC-1 cells. The C allele of rs36115365 binds specific protein complexes (arrows) that are competed by the ZNF148 antibody but not by the ZNF740 antibody. The amounts of antibodies used were 1 and 2 mg, respectively and denoted by gradient symbols. IgG was used as a negative control antibody at 2 mg per reaction.

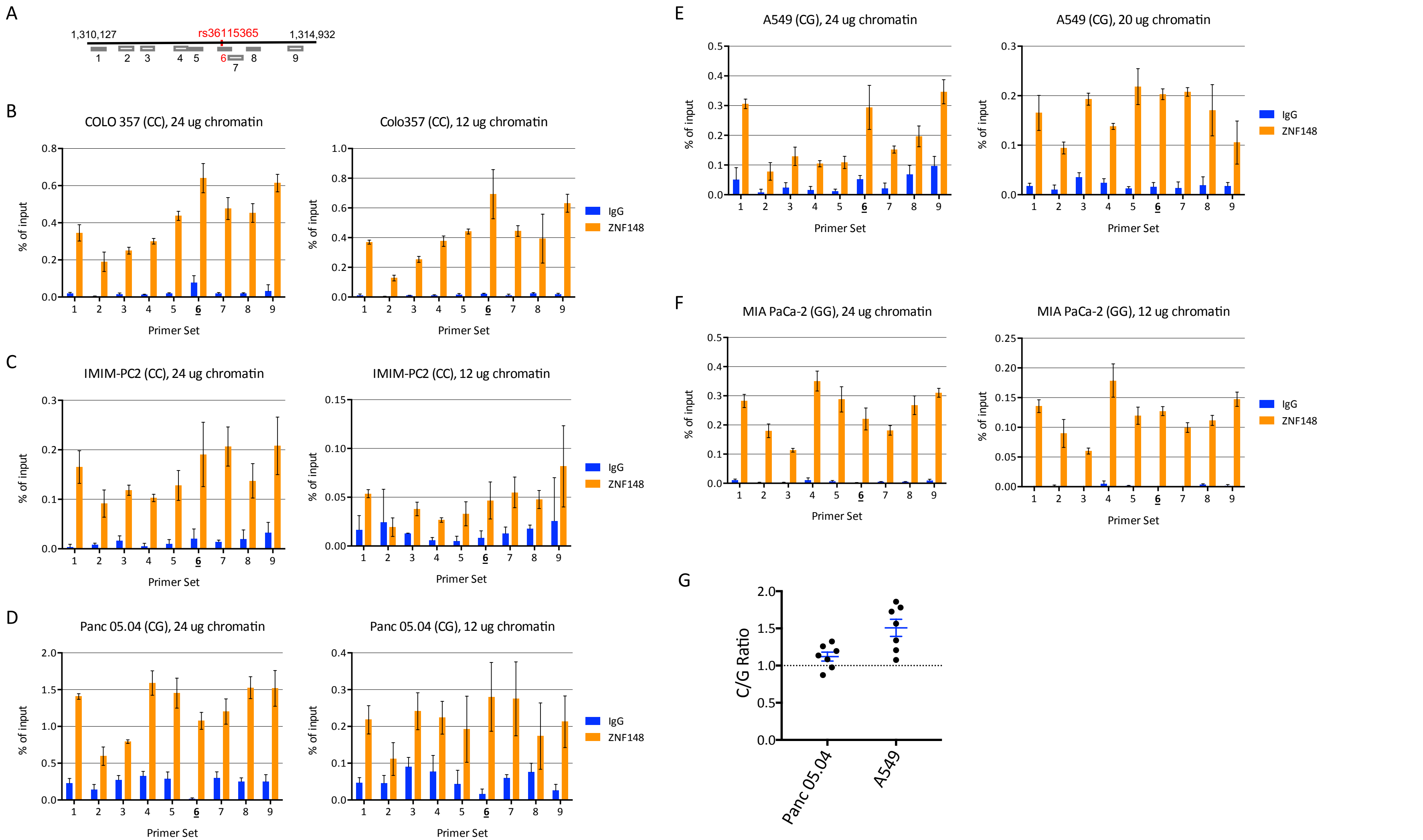

Supplementary Figure 12 | Chromatin Immunoprecipitation PCR (ChIP-PCR) for ZNF148 at rs36115365. a. ZNF148 ChIP was performed followed by qPCR of nine target regions on 5p15.33 (1,310,127-1,314,932 bp) that overlap (amplicon #6) and surround rs36115365. **a**, Map of PCR amplicons used for ChIP-PCR. Cell lines tested were homozygous (**b**, COLO 357 and **c**, IMIM-PC2) or heterozygous (**d**, Panc 05.04 and **e**, A549) for the C allele at rs36115365, or homozygous for the alternative G allele (**f**, MIA PaCa-2). Data is shown as percent enrichment over input chromatin for ZNF148 (orange bars) and control IgG (blue bars) antibodies for two independent ChIP experiments conducted using 12, 20, and/or 24ug chromatin, respectively. Experiments were conducted in triplicate; error bars represent standard deviation (SD). **g**, Allelic qPCR for rs36115365 was performed following ZNF148 ChIP using A549 (left) and Panc 05.04 cells. C- and G-allele signals are normalized to those generated from input chromatin. Data shown are from seven independent ChIP experiments; error bars represent standard error of the mean (SEM).

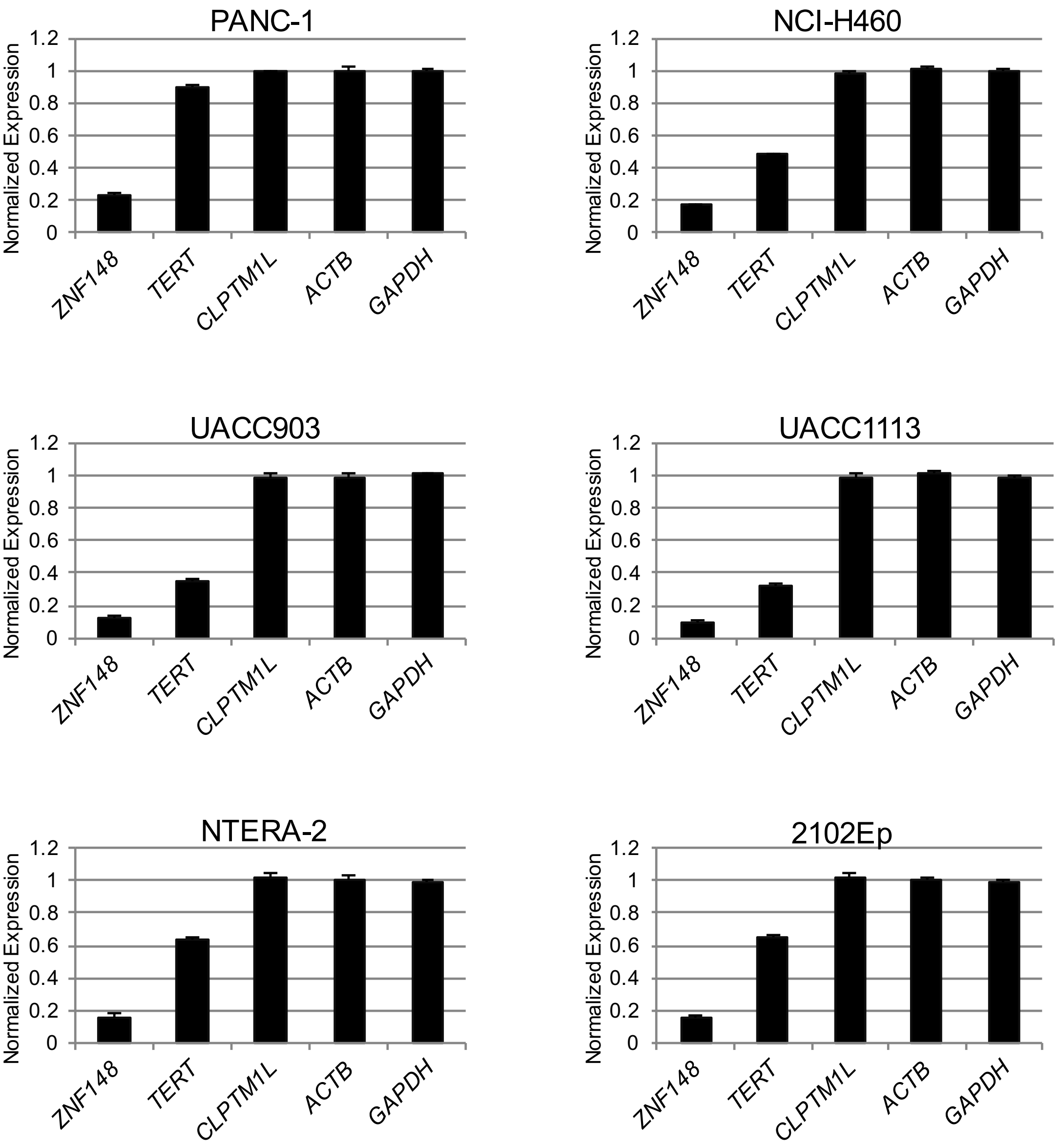

Supplementary Figure 13 | siRNA-mediated depletion of *ZNF148* reduces gene expression of *TERT* but not *CLPTM1L*. An siRNA directed against *ZNF148* transcript was transfected into PANC-1 (pancreas, upper left), NCI-H460 (lung, upper right), UACC903 (melanoma, middle left), UACC1113 (melanoma, middle right), NTERA-2 (testis, lower left), or 2102Ep (testis, lower right) cell lines, and expression of *ZNF148*, *TERT*, *CLPTM1L*, *ACTB*, and *GAPDH* were assayed by quantitative PCR. Depletion of *ZNF148* resulted in consistent reduction of *TERT* but not *CLPTM1L* or control gene expression across the majority of cell lines. Expression values were normalized to those from cells transfected with a scrambled control siRNA. Experiments were conducted in triplicate and repeated three times; error bars represent standard error of the mean (SEM) across three separate experiments.

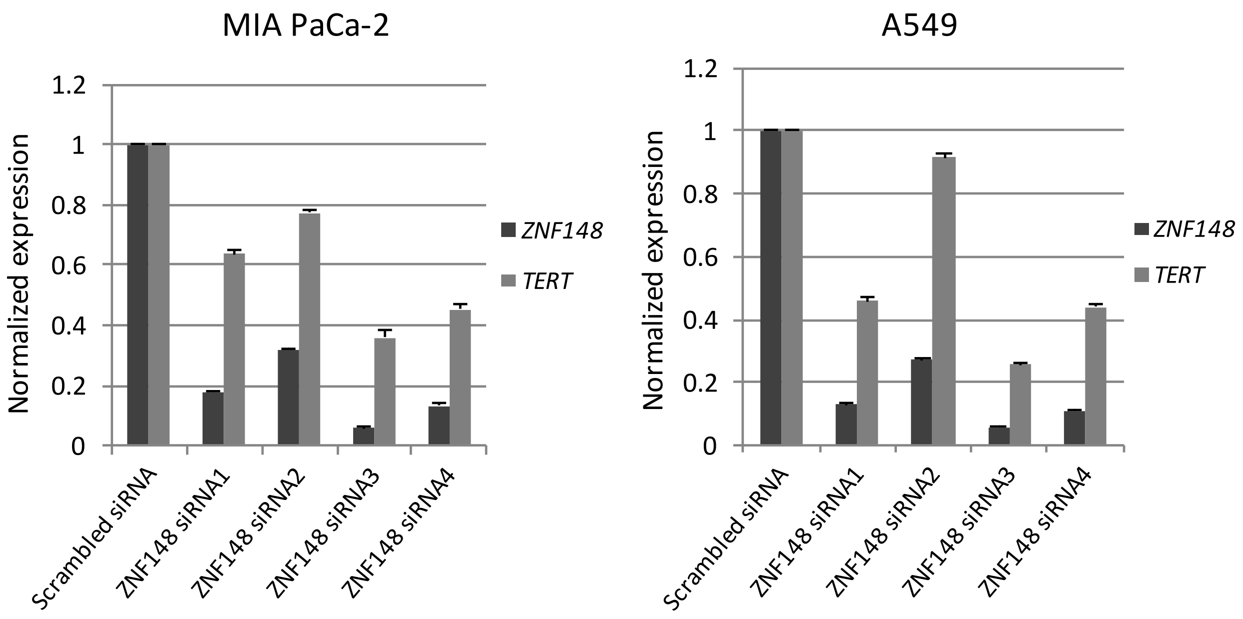

Supplementary Figure 14 | Effect of four individual *ZNF148*-targeting siRNAs on *TERT* expression. Four separate siRNAs targeting *ZNF148* were transfected into MIA PaCa-2 or A549 cells, and expression of *ZNF148* and *TERT* were measured by quantitative PCR. Expression values were normalized to those from cells transfected with a scrambled control siRNA. Experiments were conducted in triplicate and repeated three times; error bars represent standard error of the mean (SEM) across three separate experiments.

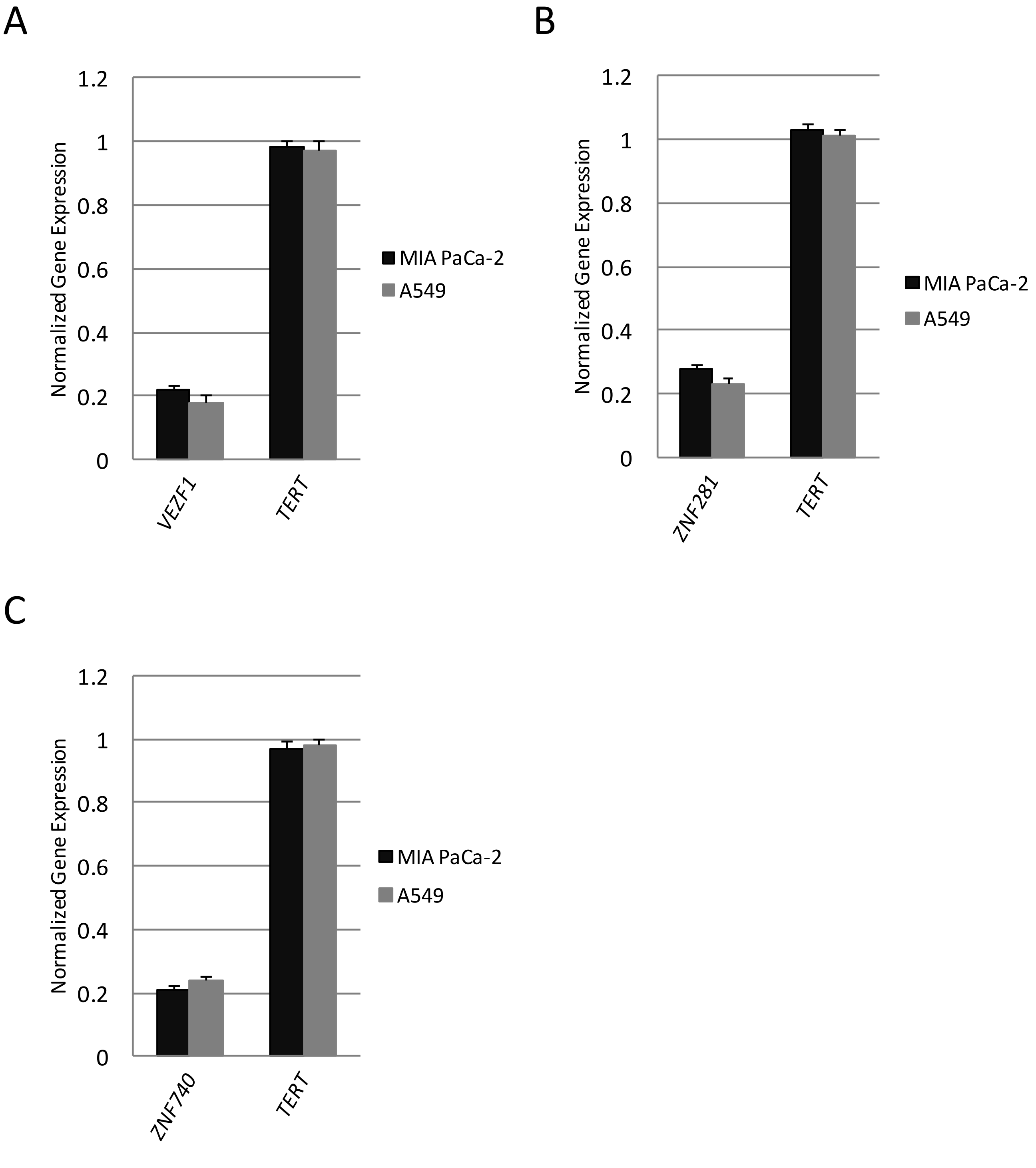

Supplementary Figure 15 | Knockdown of *VEZF1* (ZNF161), *ZNF281,* or *ZNF740* does not alter *TERT* expression. siRNAs targeting (**a**) *VEZF1* (ZNF161), (**b**) *ZNF281*, or (**c**) *ZNF740* transcripts were transfected into MIA PaCa-2 and A549 cell lines, respectively, and the expression of each gene and *TERT* were assayed by quantitative RT-PCR. Depletion of all three genes had no significant effect on the expression of *TERT*. Expression values were normalized to those from cells transfected with a scrambled control siRNA. Experiments were conducted in triplicate and repeated three times; error bars represent standard error of the mean (SEM) across three separate experiments.

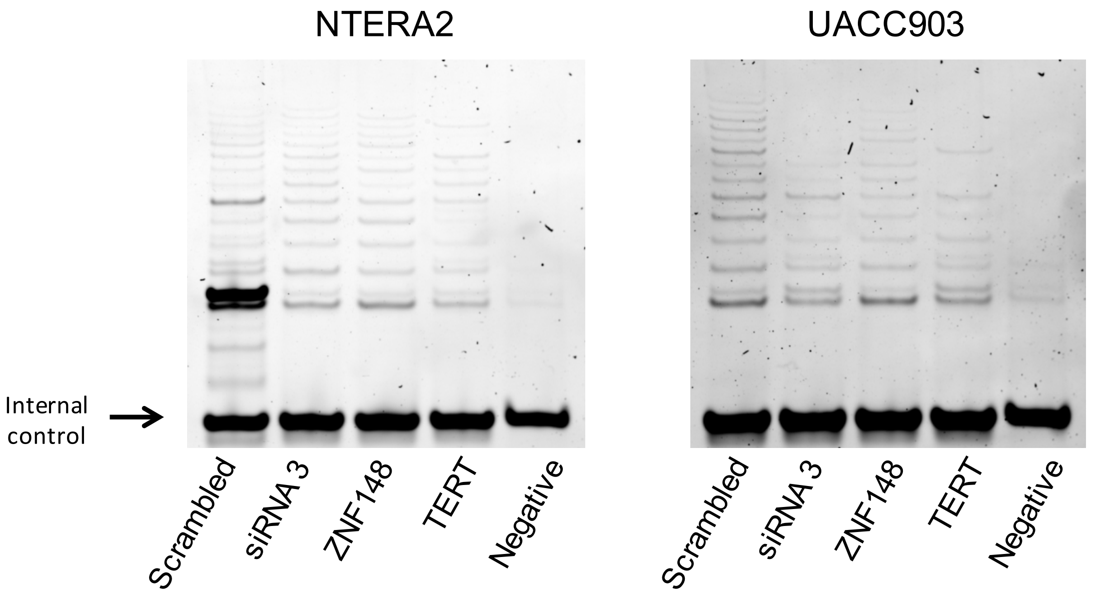

Supplementary Figure 16 | TRAP assay (telomeric repeat amplification protocol) results for testicular and melanoma cell lines. The TRAP assay was used to evaluate telomerase activity after knockdown of *ZNF148* and *TERT* by siRNA mediated post-transcriptional gene silencing (PTGS), as well as after siRNA mediated transcriptional gene silencing (TGS) of the gene regulatory element wherein rs36115365 resides via siRNA3. Scrambled: scrambled control siRNA; negative is the TRAP assay performed with no cell extract added.

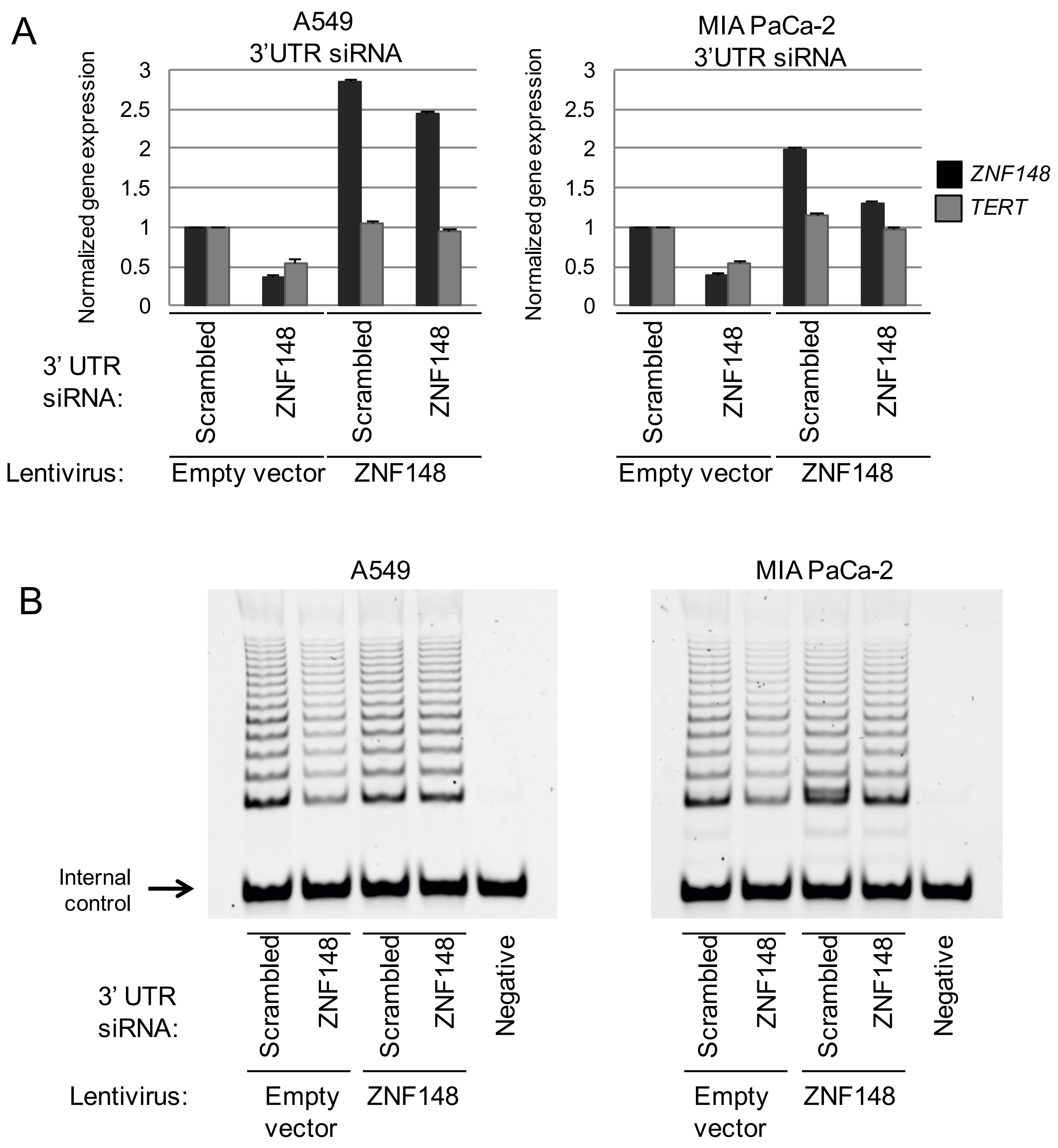

Supplementary Figure 17 | Rescue of *TERT* expression and telomerase activity in A549 (lung) and MIA PaCa-2 (pancreatic) cancer cells. siRNA targeting the 3'UTR of ZNF148, or a scrambled siRNA control, were transfected into A549 (lung, left) and MIA PaCa-2 (pancreas, right) cell lines and ZNF148 expression was rescued via lentiviral transduction. **a,** *ZNF148* (black bars) and *TERT* (grey bars) expression were measured using quantitative PCR. Experiments were conducted in triplicate and repeated three times; error bars represent standard error of the mean (SEM). **b**, Telomerase activity was measured using a telomeric repeat amplification protocol (TRAP). Negative represents the TRAP assay performed with no cell extracts added; internal control represents a 36 bp internal standard.

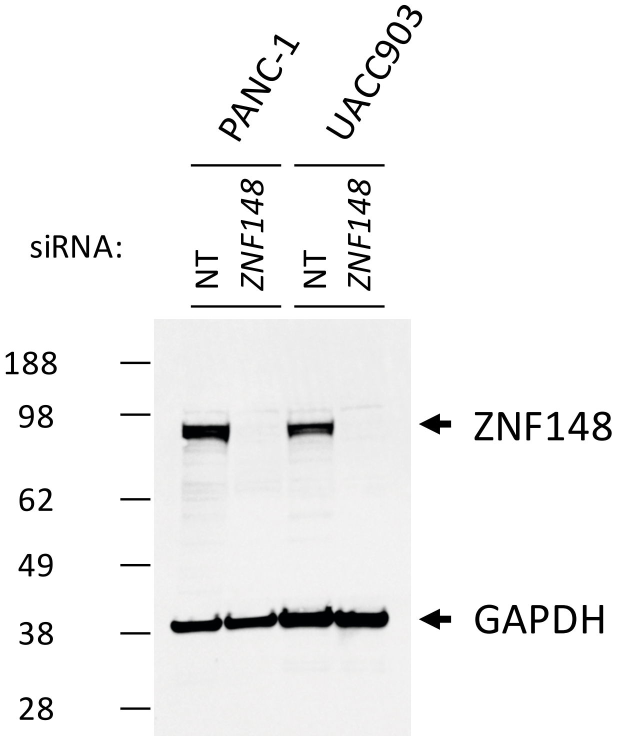

Supplementary Figure 18 | Specificity of the ZNF148 antibody. The specificity of the ZNF148 antibody was tested by western blot analysis with and without (NT, non-targeting) siRNA mediated knock-down of ZNF148 in PANC-1 and UACC903 cells. Western blot was performed using a ZNF148 antibody (upper bands). Loading control was GAPDH (bottom bands).

Supplementary Data Table 1. Association results for nine SNPs on chr5p15.33 marking "Region 2" in *CLPTM1L* in pancreatic cancer, testicular germ cell tumors, lung cancer and melanoma

Pancreatic cancer

SNP	Chr	Location	Group	Info	Subjects		Allele		MAF		OR	*P*-value	Conditional *P*-value*	
					Control	Case	Ref	Minor	Control	Case			rs451360	rs36115365
rs7446461	5p15.33	1306521	PanScan I&II	0.851	3641	3524	G	C	0.158	0.187	1.26 (1.20-1.33)	1.10x10^{-6}	0.47	0.25
rs35953391	5p15.33	1312329	PanScan I&II	0.961	3641	3524	C	T	0.195	0.235	1.29 (1.23-1.34)	1.59x10^{-9}	0.60	0.99
rs36115365	5p15.33	1313242	PanScan I&II	0.961	3642	3524	G	C	0.195	0.236	1.29 (1.23-1.34)	1.57x10^{-9}	0.60	1.00
rs13170453	5p15.33	1317481	PanScan I&II	0.940	3641	3524	A	G	0.214	0.257	1.29 (1.24-1.34)	4.36x10^{-10}	0.80	0.09
rs451360	5p15.33	1319680	PanScan I&II	0.959	3641	3524	C	A	0.215	0.259	1.29 (1.24-1.35)	2.00x10^{-10}	1.00	0.04
rs380145	5p15.33	1328897	PanScan I&II	0.946	3641	3524	C	T	0.216	0.259	1.29 (1.24-1.34)	4.33x10^{-10}	0.83	0.08
rs27071	5p15.33	1346081	PanScan I&II	0.936	3641	3524	T	C	0.253	0.297	1.28 (1.23-1.33)	6.36x10^{-10}	0.68	0.03
rs27068	5p15.33	1347239	PanScan I&II	0.932	3641	3524	C	T	0.254	0.297	1.27 (1.22-1.32)	1.08x10^{-9}	0.91	0.04
rs37004	5p15.33	1356684	PanScan I&II	0.820	3641	3524	C	T	0.224	0.264	1.31 (1.26-1.37)	3.65x10^{-10}	0.50	0.05

Testicular Germ Cell Tumors

SNP	Chr	Location	Group	Info	Subjects		Allele		MAF		OR	*P*-value	Conditional *P*-value*	
					Control	Case	Ref	Minor	Control	Case			rs35953391	rs36115365
rs7446461	5p15.33	1306521	NCI	0.911	1055	581	G	C	0.174	0.228	1.50 (1.22-1.84)	9.04x10^{-5}		
			PENN	0.829	177	477	G	C	0.141	0.193	1.61 (1.13-2.31)	8.80x10^{-3}		
			**Combined**		**1232**	**1058**					**1.53 (1.28-1.82)**	**2.62x10^{-6}**	0.21	0.22
rs35953391	5p15.33	1312329	NCI	0.977	1055	581	C	T	0.205	0.293	1.66 (1.39-2.00)	4.91x10^{-8}		
			PENN	0.955	177	477	C	T	0.180	0.244	1.53 (1.13-2.07)	5.71x10^{-3}		
			**Combined**		**1232**	**1058**					**1.63 (1.39-1.90)**	**1.08x10^{-9}**	1.00	0.92
rs36115365	5p15.33	1313242	NCI	0.977	1055	581	G	C	0.205	0.293	1.66 (1.38-1.99)	5.29x10^{-8}		
			PENN	0.956	177	477	G	C	0.180	0.244	1.53 (1.13-2.07)	5.52x10^{-3}		
			**Combined**		**1232**	**1058**					**1.63 (1.39-1.90)**	**1.10x10^{-9}**	0.92	1.00
rs13170453	5p15.33	1317481	NCI	0.974	1055	581	A	G	0.229	0.319	1.61 (1.35-1.92)	1.22x10^{-7}		
			PENN	0.953	178	477	A	G	0.202	0.270	1.51 (1.14-2.01)	4.68x10^{-3}		
			**Combined**		**1233**	**1058**					**1.58 (1.36-1.84)**	**2.11x10^{-9}**	0.37	0.36
rs451360	5p15.33	1319680	NCI	0.975	1055	581	C	A	0.228	0.313	1.57 (1.32-1.87)	5.53x10^{-7}		
			PENN	0.956	177	477	C	A	0.200	0.269	1.51 (1.13-2.01)	5.03x10^{-3}		
			**Combined**		**1232**	**1058**					**1.55 (1.34-1.80)**	**9.86x10^{-9}**	0.66	0.66
rs380145	5p15.33	1328897	NCI	0.979	1055	581	C	T	0.230	0.318	1.57 (1.32-1.88)	4.12x10^{-7}		
			PENN	0.963	177	477	C	T	0.206	0.272	1.49 (1.12-1.99)	6.08x10^{-3}		
			**Combined**		**1232**	**1058**					**1.55 (1.34-1.80)**	**8.98x10^{-9}**	0.71	0.70
rs27071	5p15.33	1346081	NCI	0.938	1055	581	T	C	0.268	0.357	1.55 (1.30-1.83)	5.67x10^{-7}		
			PENN	0.972	177	477	T	C	0.246	0.313	1.48 (1.12-1.94)	5.41x10^{-3}		
			**Combined**		**1232**	**1058**					**1.53 (1.32-1.77)**	**1.09x10^{-8}**	0.30	0.29
rs27068	5p15.33	1347239	NCI	0.934	1055	581	C	T	0.268	0.358	1.55 (1.30-1.83)	5.96x10^{-7}		
			PENN	0.973	177	477	C	T	0.246	0.313	1.48 (1.12-1.94)	5.50x10^{-3}		
			**Combined**		**1232**	**1058**					**1.53 (1.32-1.76)**	**1.15x10^{-8}**	0.31	0.30
rs37004	5p15.33	1356684	NCI	0.871	1055	581	C	T	0.240	0.314	1.53 (1.27-1.84)	5.95x10^{-6}		
			PENN	0.869	177	477	C	T	0.227	0.270	1.37 (1.02-1.84)	3.63x10^{-2}		
			**Combined**		**1232**	**1058**					**1.48 (1.27-1.74)**	**7.43x10^{-7}**	0.40	0.41

Lung cancer

SNP	Chr	Location	Group	Info	Subjects		Allele		MAF	OR	*P*-value	Conditional *P*-value*	
					Control	Case	Ref	Minor	Overall			rs37004	rs36115365
rs7446461	5p15.33	1306521	TRICL	0.824	16838	12160	G	C	0.163	0.85 (0.81-0.89)	$5.71x10^{-10}$	0.31	0.11
rs35953391	5p15.33	1312329	TRICL	0.958	16838	12160	C	T	0.191	0.87 (0.83-0.91)	$4.80x10^{-10}$	0.37	NA[#]
rs36115365	5p15.33	1313242	TRICL	0.958	16838	12160	G	C	0.191	0.87 (0.83-0.91)	$4.76x10^{-10}$	0.37	1.00
rs13170453	5p15.33	1317481	TRICL	0.954	16838	12160	A	G	0.213	0.85 (0.82-0.89)	$7.62x10^{-13}$	0.09	$2.97x10^{-4}$
rs451360	5p15.33	1319680	TRICL	0.955	16838	12160	C	A	0.211	0.85 (0.82-0.89)	$8.50x10^{-13}$	0.09	$3.44x10^{-4}$
rs380145	5p15.33	1328897	TRICL	0.955	16838	12160	C	T	0.213	0.86 (0.82-0.89)	$1.46x10^{-13}$	0.23	$7.71x10^{-4}$
rs27071	5p15.33	1346081	TRICL	0.891	16838	12160	T	C	0.251	0.86 (0.84-0.88)	$7.93x10^{-12}$	0.45	$2.41x10^{-3}$
rs27068	5p15.33	1347239	TRICL	0.888	16838	12160	C	T	0.252	0.86 (0.84-0.88)	$6.26x10^{-12}$	0.42	$1.95x10^{-3}$
rs37004	5p15.33	1356684	TRICL	0.853	16838	12160	C	T	0.224	0.84 (0.81-0.88)	$1.18x10^{-13}$	1.00	$3.74x10^{-5}$

Melanoma

SNP	Chr	Location	Group	Info	Subjects		Allele		MAF		OR	*P*-value	Conditional *P*-value*	
					Control	Case	Ref	Minor	Control	Case			**rs2447853**	**rs36115365**
rs7446461	5p15.33	1306521	GenoMEL	0.908	7691	5374	G	C	0.173	0.171	0.98 (0.92-1.05)	0.62	$5.77x10^{-5}$	0.09
rs35953391	5p15.33	1312329	GenoMEL	0.959	7691	5374	C	T	0.207	0.209	1.01 (0.95-1.08)	0.72	$1.04x10^{-4}$	0.22
rs36115365	5p15.33	1313242	GenoMEL	0.959	7691	5374	G	C	0.207	0.209	1.01 (0.95-1.08)	0.70	$1.09x10^{-4}$	1.00
rs13170453	5p15.33	1317481	GenoMEL	0.960	7691	5374	A	G	0.228	0.236	1.04 (0.98-1.11)	0.18	$1.48x10^{-3}$	$9.31x10^{-3}$
rs451360	5p15.33	1319680	GenoMEL	0.960	7691	5374	C	A	0.227	0.235	1.05 (0.99-1.11)	0.14	$2.47x10^{-3}$	$5.63x10^{-3}$
rs380145	5p15.33	1328897	GenoMEL	0.961	7691	5374	C	T	0.231	0.238	1.04 (0.98-1.11)	0.18	$1.06x10^{-3}$	0.01
rs27071	5p15.33	1346081	GenoMEL	0.935	7691	5374	T	C	0.275	0.281	1.04 (0.98-1.10)	0.19	$2.69x10^{-3}$	0.073
rs27068	5p15.33	1347239	GenoMEL	0.937	7691	5374	C	T	0.276	0.282	1.04 (0.98-1.10)	0.19	$2.49x10^{-3}$	0.076
rs37004	5p15.33	1356684	GenoMEL	0.871	7691	5374	C	T	0.240	0.248	1.05 (0.99-1.12)	0.10	$4.45x10^{-3}$	$5.98x10^{-3}$

Conditional analysis was performed for the most significant SNP in Region 2 for each cancer as well as for rs36115365. Info: IMPUTE2 imputation INFO score for pancreatic cancer, testicular cancer and melanoma; the weighed average of INFO and RSQ scores for lung cancer based on sample size in the individual TRICL studies. Overall minor allele frequency was available for lung cancer. NA#: the high LD between rs36115365 and rs35953391 do not allow for results for the latter in the conditional analysis using TCGA.

Supplementary Table 2: Association results for variants in 1000G Phase 3 version 1 for pancreatic cancer

SNP	OR (95% CI)	*P*	Info	Conditional analysis		EUR MAF	r^2
				$P_{\text{Conditioning on rs36115365}}$	$P_{\text{Conditioning on SNP in first column}}$ for rs36115365		
rs539580303	1.30 (1.13-1.15)	2.45×10^{-4}	0.85	0.99	2.13×10^{-3}	0.093	0.44
rs551404639	1.30 (1.13-1.15)	2.45×10^{-4}	0.85	0.99	2.13×10^{-3}	0.093	0.44
rs116309746	1.30 (1.06-1.60)	0.011	0.84	0.83	8.23×10^{-5}	0.037	0.16
rs71575564	1.18 (1.10-1.26)	1.03×10^{-6}	0.99	0.02	6.78×10^{-3}	0.443	0.27
rs3030832	1.28 (1.18-1.39)	8.25×10^{-10}	0.98	0.06	0.80	0.214	0.87
rs531693130	1.42 (1.19-1.71)	9.8×10^{-5}	0.79	0.04	1.03×10^{-5}	0.074	0.02
rs115251750	1.36 (1.24-1.49)	8.3×10^{-4}	0.91	0.09	2.91×10^{-4}	0.036	0.13
rs148487301	1.41 (1.27-1.57)	8.0×10^{-4}	0.98	0.03	3.89×10^{-5}	0.031	0.13

Association results for pancreatic cancer for 8 variants from 1000G Phase 3 version 1 that had greater OR than rs36115365 or *P* values$<5 \times 10^{-6}$. Conditional analysis was performed for each of the SNPs in the first column as well as for rs36115365. Info: IMPUTE2 imputation INFO score. EUR MAF is minor allele frequency in 1000G EUR populations in Phase 3, version 1. r^2 is correlation to rs36115365 in the 1000G EUR populations.

Supplementary Table 3. Oligos used in EMSA experiments.

SNP	Chr	Location (Hg19)	Forward Oligo	Reverse Oligo
rs35953391	5p15.33	1,312,329	GACGGCCATGTACAAG(C/T)CAAGGAGAGGCTGAC	GTCAGCCTCTCCTTG(G/A)CTTGTACATGGCCGTC
rs36115365	5p15.33	1,313,242	GGTCTCAGCCTCACC(G/C)TCCGTGGCCACGGCAG	CTGCCGTGGCCACGGA(C/G)GGTGAGGCTGAGACC
rs13170453	5p15.33	1,317,481	GATCACTTGAGATCAGG(A/G)GTTCGAGACCAGCCTG	CAGGCTGGTCTCGAAC(T/C)CCTGATCTCAAGTGATC
rs451360	5p15.33	1,319,680	GGTCCGGGGTGCCG(G/T)CCCTTGGGGAGCGCAG	CTGCGCTCCCCAAGGG(C/A)CGGCACCCCGGACC
rs380145	5p15.33	1,328,897	GAAATGTGTCTGTAGAG(G/A)AGGATCTGGATGGAGTGTC	GACACTCCATCCAGATCCT(C/T)CTCTACAGACACATTTC
rs37004	5p15.33	1,356,684	CAAGAACCCTCTCTTG(G/A)GTTCTGGATCAGGAC	GTCCTGATCCAGAAC(C/T)CAAGAGAGGGTTCTTG
rs7446461	5p15.33	1,359,521	CTCCTGTACTCAAGA(G/C)ATCCTCCCACCTCAC	GTGAGGTGGGAGGAT(C/G)TCTTGAGTACAGGAG
rs3030832	5p15.33	1,388,386	GGCTGACCTGGGGCCACA(CA/--)GGGATGGGAAGGGGGATG	CATCCCCCTTCCCATCCC(TG/--)TGTGGCCCCAGGTCAGCC
rs27071	5p15.33	1,399,081	CCATCCGCCCGTCAGCC(A/G)AGTCCTCTGAGTTCTTCC	GGAAGAACTCAGAGGACT(T/C)GGCTGACGGGCGGATGG
rs27068	5p15.33	1,400,239	GTTTGAGGAGTTCTGCG(G/A)GGTGCTGAATGGGGTCCTC	GAGGACCCCATTCAGCACC(C/T)CGCAGAACTCCTCAAAC
ZNF148 binding motif*	6p21.2	36,646,269	CGCAGGCGAGGGACTGGGGGAGGAGGGAAGTGCCC	GGGCACTTCCCTCCTCCCCCAGTCCCTCGCCTGCG

*ZNF148 binding motif is from a known ZNF148 binding site in the *CDKN1A/p21* promoter

Supplementary Table 4. Primer Sequences Used for ZNF148 ChIP

Name	Chr	Location (Hg19)	Forward	Reverse
ZNF148_1	5p15.33	1,310,363 - 1,310,587	GCCCAGTTTCCACACAGTTC	GATCCTGAAGCTCAGCCAA G
ZNF148_2	5p15.33	1,310,973 - 1,311,173	CCCCAAAGTGGGGCTTAG	ACTTGCTTGAGCCCACTCC
ZNF148_3	5p15.33	1,311,386 - 1,311,582	CAGGAGGGGACTCATCAGAG	GGCAGGAGAATAGGGTCTG G
ZNF148_4	5p15.33	1,312,213 - 1,312,430	GTGGCCCTAAGCCAGTGAC	ATTGGCCAACTCAGTTCTGG
ZNF148_5	5p15.33	1,312,417 - 1,312,583	CTGAGTTGGCCAATGTCTGC	CCTGGCCCAAATATCAACTG
ZNF148_6	5p15.33	1,313,216 - 1,313,399	CAGGAGGAAATGGTCTCAGC	GCATTTCCCTGCTGACTAGC
ZNF148_7	5p15.33	1,313,370 - 1,313,539	TTTCAACGCCGCTAGTCAG	CTAAGGGGTCTGTGCCACTC
ZNF148_8	5p15.33	1,313,706 - 1,313,910	TTGCGACAACACAGATGGAC	ATGCTTCCAGACTCCAGCAC
ZNF148_9	5p15.33	1,314,715 - 1,314,920	GACTCCTGGCCTCCAGAAC	GCTTACCCCTTTCCTGAAGC

Supplementary Table 5. Primers for cloning the genomic region surrounding rs36115365 into pGL4.23 for luciferase assays

SNP	Chr	Location (Hg19)	Forward Oligo*	Reverse Oligo*
Forward clone	5p15.33	1,313,140 - 1,313,379	TAATGCTAGCCAAAGGAAACCGCAGACTTAG	TAATAGATCTGGCGTTGAAAACCCTTTGTATC
Reverse clone	5p15.33	1,313,140 - 1,313,379	TAATGCTAGCGGCGTTGAAAACCCTTTGTATC	TAATAGATCTCAAAGGAAACCGCAGACTTAG

*The NheI and BglII restriction enzyme sites used for cloning are underlined (NheI: GCTAGC; BglII: AGATCT) for each oligonucleotide. Forward orientation is the same as the genomic orientation.

Supplementary Table 6. Sequences of double-stranded siRNA for targeting the region surrounding rs36115365.

Name	Chr	Location (Hg19)	Sense	Antisense
Control siRNA	-	-	UGGUUUACAUGUCGACUAA	UUAGUCGACAUGTAAACCA
siRNA1	5p15.33	1,313,141-1,313,159	AAAGGAAACCGCAGACUUA	UAAGUCUGCGGUUUCCUUU
siRNA2	5p15.33	1,313,223 - 1,313,241	GGUGAGGCUGAGACCAUUU	AAAUGGUCUCAGCCUCACC
siRNA3	5p15.33	1,313,251 - 1,313,269	GCUCACUGAAGCUGCCGUG	CACGGCAGCUUCAGUGAGC
siRNA4	5p15.33	1,313,368 - 1,313,386	GACUAGCGGCGUUGAAAAC	GUUUUCAACGCCGCUAGUC
siRNA5	5p15.33	1,313,463 - 1,313,481	AUUGUGUGCUGGAGAAGGG	CCCUUCUCCAGCACACAAU
siRNA6	5p15.33	1,313,536 - 1,313,554	UUAGAACACGGGUGACAUG	CAUGUCACCCGUGUUCUAA
siRNA7	5p15.33	1,313,655 - 1,313,673	CCGGUUCGUGCCAUGGAAU	AUUCCAUGGCACGAACCGG
siRNA8	5p15.33	1,313,704 - 1,313,722	GCUUGCGACAACACAGAUG	CAUCUGUGUUGUCGCAAGC
siRNA_8q24_1	8q24.1	128,408,433 - 128,408,451	CAGCAAACAUUCACAGGCA	UGCCUGUGAAUGUUUGCUG
siRNA_8q24_2	8q24.1	128,413,315 - 128,413,353	UCUCAGUGCCUUUCAUCUG	CAGAUGAAAGGCACUGAGA
siRNA_8q24_3	8q24.1	128,519,850 - 128,519,868	CCAUAGAUAAUACGUAAAC	GUUUACGUAUUAUCUAUGG
siRNA_8q24_4	8q24.1	128,413,254 - 128,413,272	UCUUAGUGGUAGGAGGAGA	UCUCCUCCUACCACUAAGA

The ON-TARGET-Enhanced Antisense Loading siRNAs were purchased from Dharmacon in GE Healthcare.
